# Multi-variate coding for possession: methodology and preliminary results

**DOI:** 10.1515/ling-2022-0021

**Published:** 2023-11-09

**Authors:** Natalia Chousou-Polydouri, David Inman, Thomas C. Huber, Balthasar Bickel

**Affiliations:** Department of Comparative Language Science and Center for the Interdisciplinary Study of Language Evolution, University of Zurich, Zürich, Switzerland

**Keywords:** database, inalienable, methodology, non-possessible, possession

## Abstract

In this work we are presenting a database structure to encode the phenomenon of differential possession across languages, considering noun possession classes and possessive constructions as independent but linked. We show how this structure can be used to study different dimensions of possession: semantics, noun valence, and possessive constructions. We present preliminary survey results from a global sample of 120 languages and show that there is a universal semantic core in both inalienable and non-possessible noun classes. Inalienables are centered on body parts and kinship. Non-possessibles are centered on animals, humans, and natural elements.

## 1 Introduction

The phenomenon of possession, and especially differential possession, has a lengthy pedigree of linguistic investigation. One of the earliest mentions of the phenomenon is in Lévy-Bruhl’s (1914) description of possession in Melanesian languages, where he associates different possessive strategies with different constructional or lexical semantics. Distinctions among noun possession classes have enjoyed a sustained period of linguistic interest ever since, both in language description and in theoretical and typological studies (Bickel and Nichols 2013; Bugaeva et al. 2021; Chappell and McGregor 1989; Haspelmath 2017; Nichols 1988; Seiler 1983; Stolz et al. 2008). This line of investigation into possessive splits is complemented by more general typologies of possessive morphosyntax (Koptjevskaja-Tamm 2008).

There is however a descriptive gap in framing differential possession as an issue of (in)alienability, driven especially by data from the Americas, namely the existence in some languages of non-possessible nouns (Bickel and Nichols 2013). For example, in K’iche’ (Mayan), in addition to multiple inalienable classes, there is a class of nouns expressing natural elements which cannot be directly possessed, but must be possessed through a metaphorical (and inalienable) kin relation.

(1)

**Table:** 

*q-ati’t*	*iik’*	
1pl-grandmother	moon	
‘our grandmother moon’ (our moon)		
(López Ixcoy 1997: 104, glossing added)		(K’iche’, Mayan)

The addition of a third member to the traditional alienable-versus-inalienable distinction is reason enough to revisit the typologization of possession. It is becoming clear that any full account of differential possession should also include the opposite of inalienables: super-alienables or “non-possessibles” that are in some way syntactically or semantically “difficult” to possess (Haspelmath 2017), the same way that inalienable nouns are “easy” to possess (or “difficult” to unpossess).

Even though alienability distinctions are a highly researched area of linguistic study, we see two issues of comparability when reviewing prior typological work. The first is that researchers often investigate different aspects of possession, and in a way that makes their results not easy to compare or synthesize. The second difficulty, which is less serious on its own but compounds the first, is that researchers often use the same terminology, explicitly or implicitly, to describe different grammatical phenomena.

One way to approach possession is to study the morphosyntactic permissibility and possibility of adnominal possession, i.e., whether particular nouns are *obligatorily possessed*, *optionally possessed*, or *non-possessible* (Bickel and Nichols 2013; Bugaeva et al. 2021). Another approach investigates the syntactic differentiation between a lexically open or default class of nouns and one or more lexically specified classes of nouns (often assumed to involve “inalienable” semantics, as in Seiler 1983). These two views are related, as a category of obligatorily possessed nouns is virtually guaranteed to be a non-default category. However, possession type is not always directly associated with a class being default or non-default. A default class could be non-possessible or optionally possessed. A non-default class, on the other hand, could fall into any of the three morphosyntactic categories.

A typological paper on differential possession which pre-supposes a semantic component to the split (Haspelmath 2017), a paper investigating different morphosyntactic constructions of possession regardless of the presence of an alienability split (Koptjevskaja-Tamm 2008), and a paper investigating the (dis)obligation of adnominal possession (Bugaeva et al. 2021) are intuitively all looking at “the same phenomenon.” However, because they approach it from different angles, it is difficult to directly compare what these researchers have discovered and to appreciate the typological variation of possession expressions.

The second issue is the implicit definitions present behind terms like “inalienable” and “alienable”. Researchers investigating other aspects of possession often take the meanings of these terms to be already established (Barker 2011; Bickel and Nichols 2013; Bugaeva et al. 2021; Chappell and McGregor 1989). Nichols (1988) makes this explicit, stating that, for her sample, there is “never any doubt as to which member […] will be called *inalienable* and which will be called *alienable*.” As with concepts like “transitive subject” and “object”, this assumption is made not because it would be impossible to precisely ground them, but because it is not always the most relevant matter in a given research program, and the definitions can be taken as established elsewhere in linguistic science. Most typically for typological work, the inalienable class is implicitly assumed to have both semantic and syntactic features: semantically to include the notion of “inherent” possession, and syntactically to acquire special, non-default possessive marking.

Instead of an implicit definition, Haspelmath (2017) explicitly defines the inalienable category as that which includes kinship terms, body parts, or both. There are still other uses of the terms in which the inalienable nouns are taken to be equivalent to obligatorily possessed nouns, or sometimes to carry a different kind of lexical valence or argument structure (Alexiadou 2003).

This suite of definitions, both implicit and explicit, means that the “alienable” and “inalienable” terminology is used in different ways in different grammars or descriptive traditions. Some writers discuss alienability even when a language lacks any grammatical correlates for the distinction (as one finds occasionally in descriptive work, e.g., McGregor 1990: 253). Other authors make statements such as “no alienable and inalienable distinction is made” (in a particular language) (Walsh 1976: 281). Though not always made clear, there are two possible interpretations of such statements, which we believe are insufficiently distinguished: (1) that conceptual possessive distinctions exist in all languages, but whether they are grammatically encoded or not depends on the particularities of the language; and (2) that the conceptualization of possession itself varies between languages. Though these two views might be practically equivalent when describing a language’s grammar, moving between different notions of (in)alienability can conceal presuppositions about the universality versus particularity of such distinctions. It is worthwhile, in our view, to make explicit the semantic and/or syntactic components of (in)alienability.

### 1.1 Scope

Though the focus of the present study is differential possession and not possession in a broader sense, we will nevertheless begin with a brief discussion delimiting the phenomenon of “possession”, an issue at least as vexed in the literature as the definition of “alienable” and “inalienable.” From a strictly semantic perspective, Heine (1997) delimits seven semantic possessive notions. From a much more syntactic perspective, Lehmann (1998) notes that the possession relationship is asymmetric (i.e., X poss Y ≠ Y poss X) while being essentially semantically empty, i.e., lacking lexical specification, and thus open to many semantic interpretations.

For our present investigation, we take Lehmann’s definition of possession as an asymmetric relationship between a head and a dependent noun, with the added requirement that both nouns involved be referential. This excludes constructions such as English “palm tree”, where the first noun refers to a kind, not a referent, and also excludes the expression of semantic attribution via a possessive construction.1An example of such a construction can be found in Belhare as in (1), where the grammatical possessor is in fact attributively describing the possessed noun.

(i)

**Table:** 

*khim u-choũwat*	*(*or *u-choũwat khim)*	
house 3s.poss-new		
‘(a/the) new house’		
(Bickel 2003: 563)		(Belhare, Sino-Tibetan)
This phenomenon is explored typologically in Rießler (2016).

We have not, however, followed Lehmann in requiring an empathy hierarchy for the possessor and possessed noun or the notion of a prototype. We furthermore limit our study to possession constructions in the adnominal domain and possession differences that are lexically constrained by the possessed (or un-possessed) noun.

With the theoretical scope defined, there are still many potential ways to typologically compare adnominal possession. Adnominal possession itself is multi-dimensional, relating a possessor to a possessed noun (sometimes of a particular semantic category) by means of a particular grammatical construction. Our database is structured so that, rather than choosing which point of comparison we would like to make in advance, all of these relationships can be captured at once and multiple comparisons can be made using the same database, following the principle of “late aggregation” (Witzlack-Makarevich et al. 2021). To capture the association between grammatical constructions and (un-)possessed nouns, we use the concept of a (noun) *possession class*, a collection of nouns which share a *constructional profile*, or in other words have access to the exact same set of possession (and un-possession) constructions. Appeal to a *constructional profile* is necessary because it may be the case that two nouns have access to the same possession construction, but are differentiated by their access to a second construction.

A theoretical example is given in Table 1, where two possession constructions can be used for a total of six nouns. In this situation there are three different *constructional profiles* (while only two possessive constructions): one group of nouns (represented by ‘mother’, ‘father’) has access to both constructions, while a second group (‘dog’, ‘house’) only has access to the genitive construction, and a third (‘hand’, ‘foot’) only has access to the juxtaposition construction.

**Table 1:** Theoretical example 1.

Construction	Possessed nouns
PSSR-GEN PSSD	mother, father, dog, house
PSSR PSSD	mother, father, hand, foot

In addition to possession constructions, we capture un-possession constructions, or how a noun appears when there is no possessor. In the majority of cases this is simply the bare noun. Another theoretical example is given in Table 2, which includes un-possession constructions (the bare noun and the noun marked by *ha-*) and shows a case where there are fewer possession classes (two) than possession constructions (three). One set of nouns (‘hand’, ‘foot’) has access to one possession construction and one un-possession construction, while another set (‘mother’, ‘father’, ‘dog’, ‘house’) has access to two possession constructions and one un-possession construction.

**Table 2:** Theoretical example 2.

Construction	(Un-)possessed nouns
PSSR-GEN PSSD	mother, father, dog, house
PSSR-GEN AGR-PSSD	mother, father, dog, house
PSSR PSSD	hand, foot
NOUN	mother, father, dog, house
*ha*-NOUN	hand, foot

These examples show a potentially many-to-many relationship between possession constructions and target nouns. Instead of listing every noun affiliated with a possession construction, we construct *noun possession classes*, and for each construction list which possession classes have access to it as a possessed noun. Thus, lexical information about the possession class and morphosyntactic information about the construction are cataloged independently. Though it is possible to define a possession class statistically – that is, a set of nouns may have a demonstrable preference for one construction over another, but be grammatically acceptable for both – we will only consider categorical possession classes in this paper.

#### 1.1.1 Constructions

In our database we list all possessive constructions resulting in a noun phrase, with overt nominal possessors (as opposed to pronominal possessors), and headed by the possessed noun. This excludes predicative possession constructions (like “she owns a house”) and external possession (like “she slapped me on the back”), which can behave differently from adnominal possesssion in regard to their splits (Stolz et al. 2008: 483).2Other constructions beyond the NP are also out of scope. One example is the possessive locational prefixing of Nakara (Maningrida), in which certain prefixes are only permitted with body parts and indicate both possession and location (thus resulting in an adpositional phrase), as in ‘on your head’ or ‘at (under) her arm’ (Eather 2011: 120–121). Another example is Galo (Sino-Tibetan), in which body parts and inalienable properties can enter into a type of topicalization construction where the possessor is the topic and the possessed noun is the subject (Post 2007: 711). Pronominal possession constructions are only included in two cases: (1) if nominal possessive constructions are not described or exemplified in the resources available; or (2) if two or more possession classes are distinguished only through pronominal possession (i.e., the distinction is neutralized in nominal possession).3For example, in Gooniyandi (Bunaban), all nouns can be possessed either by marking the overt possessor with dative case, or by using an oblique free pronoun. However, kinship terms have access to an additional construction when the possessor is not overt: a set of person suffixes can attach to the kinship term (McGregor 1990). Within our sample of 120 languages we found only three languages apart from Gooniyandi that also have possession classes that can only be distinguished through pronominal possession: Berta (isolate), Bilua (isolate), and Georgian (Kartvelian).


The above constraints mean that in English the constructions [PSSR*=s* PSSD] (e.g., *Mary’s car*) and [PSSD *of* PSSR] (e.g., *the car of Mary*) are within scope for our study. However, the construction [POSS.DET=PSSD] (e.g., *her car*) is out of scope, as the possessor is only indicated by a pronominal determiner. The construction [PSSD COP POSS.PRO] (e.g., *the car is hers*) is also out of scope, since it is predicative and cannot function as an NP. It is not necessarily the case though that the possession must be expressed within one noun phrase. For example, in some languages there are nouns that cannot be possessed directly, but only periphrastically within a subordinate phrase (e.g., *the land which Mary owns*) or using an appositive phrase containing a possessive classifier4Note that in a language with multiple possessive classifiers, we encode a single possessive classifier construction with a classifier slot (as opposed to a separate construction for each classifier). (e.g., *the dog, Mary’s pet*). In these cases, the end result is still an NP, and therefore is within scope for our study.

In addition to possession constructions, we also code un-possession constructions which are grammatically required when a noun is un-possessed. In some languages, the only difference between possession classes is that one set of nouns have special morphology when they are not possessed. Like the possession constructions, the un-possession constructions available to a noun are part of its constructional profile.

A consequent limitation of our current database is that an (in)alienability distinction is not captured if it is not manifested in NP constructions. In fact, there are languages, where an (in)alienability distinction is only visible in a possession construction that is outside of our scope, such as possessor raising (as can be seen in many Eurasian languages), or predicative possession. Our database design is easily extensible to include such constructions if needed.

#### 1.1.2 Possession classes

Possession classes are lexically specified sets of nouns with a unique constructional profile. Because of this definition, we sometimes distinguish more or less possession classes than a traditional linguistic description would provide. Teko (Tupian) is a good example of such a case.5For another example of our process, see Apurinã in the Supplementary Materials. The grammar of Teko recognizes three noun classes: dependent nouns, autonomous nouns, and absolute nouns (Rose 2011). The criterion for this distinction is essentially syntactic valence (something we will return to in Section 1.2.2): dependent nouns are obligatorily possessed, autonomous nouns are optionally possessed, and absolute nouns are non-possessible. However, there are two different constructions to un-possess dependent nouns and each noun seems to have access to either one or the other. At the same time, among absolute nouns, only animals can be possessed through a classifier construction. According to our criterion of a unique constructional profile, we therefore distinguish five possession classes in Teko: two of dependent nouns (differentiated by their un-possession construction), one of autonomous nouns, and two of absolute nouns (one possessible through the classifier construction and the other absolutely non-possessible).

A possession class must also be lexically specified. This means that we do not include possession classes that are phonologically conditioned. However, in our database a possession class may or may not be semantically coherent.6For examples of languages with semantically coherent possession classes, see Negidal and Amarakaeri in the Supplementary Materials. There are of course idiosyncrasies to how languages split up their nouns (e.g., for some languages “house” or “bow” are inalienable, while for others they are alienable), so we consider a class semantically coherent when nearly all members of the class fall within one or a few semantic categories (see Section 2.1.1.1) and those members comprise a substantial number of the lexical items belonging to that semantic category. A small number of additional and exceptional nouns can be included (nouns belonging to the possession class that do not fall within the semantic category). If there are a few members from many semantic categories, we consider the class semantically non-coherent.

Even though in the vast majority of cases semantic generalizations can be made, there are languages with possession classes that are highly arbitrary from a semantic point of view (e.g., see Highland Oaxaca Chontal in the Supplementary Materials).

Another scope limitation regarding possession classes is that we only take into account how nouns are categorized when they are the head of a possessive NP. There are languages where different nouns have access to different constructions when they are the possessor of a possessive NP. Such distinctions are noted in the remarks of the relevant constructions, but we have not explicitly coded for possessor classes, though such an extension of our methodology is possible.

In some languages, it is not the case that different nouns have access to different possessive constructions, but rather that the construction choice changes the meaning of the possessive relationship. In such cases, it is common that the grammar uses “alienable” and “inalienable” to refer to different constructions. An example of such a language is Krongo (Kadugli-Krongo), where e.g., alienably possessed meat (using a possessive marker) means “my meat that I bought” and inalienably possessed meat (using a genitive marker) means “my flesh, meat of my body” (2).

(2)

a.

**Table:** 

*úudà*	***kà*** *-káaw*
meat	poss-person
‘The person’s meat (bought, to be eaten)’

b.

**Table:** 

*úudà*	***má*** *-káaw*
meat	gen-person
‘The person’s flesh (part of their own body)’	
(Reh 1985: 317)

c.

**Table:** 

*òofò*	*kà-níŋ*
grave	poss-3
‘his grave (that he owns)’

d.

**Table:** 

*òofò*	*m-íˀìŋ*	
grave	gen-3	
‘his grave (in which he lies)’		
(Reh 1985: 317)		(Krongo, Kadugli-Krongo)

This is not a difference driven by possession classes (as there are no different constructional profiles across possessed nouns), but a difference in the semantics of the construction. However, a relatively common but restricted distinction of this kind is observed in many languages where body parts are inalienable: if body parts are possessed alienably, it means that they are detached from their inherent possessor. For example, “his inalienable head (still attached)” contrasts with “his alienable head (of the animal that he killed)”. In such cases, where only body parts can be used as both inalienable and alienable with a distinction in meaning,7We have not encountered a case where such a semantic distinction is present for another restricted semantic category, apart from body parts. we have only taken into account the behavior of body parts when possessed by their inherent possessor in terms of construction access, but we have noted the more “exceptional” use as a comment (for an example, see Negidal in the Supplementary Materials). However, in cases where this pattern is broader, as in Krongo, we indicate that both constructions are accessible to all nouns with a difference in meaning, but we do not consider the language as having possession classes.

### 1.2 Different comparanda of possession

We have already illustrated that there is more than one way to approach possession, depending on what one is investigating. A partial list of the grammatical properties one could target for comparison is:

– The construction(s) used to express possession
– The construction(s) used to express un-possession
– The grammatical properties of the possessed noun: definiteness, specificity, gender, mass versus count, etc.
– The grammatical properties of the possessor noun: definiteness, specificity, person, gender, etc.
– The lexical class of the possessed noun
– The semantic class of the possessed noun
– The obligation (or optionality) of expressing a possessor

Using data in the form of separate sets of constructions and possession classes, we can derive most of these comparisons. We illustrate this with a few of the commonest ways of comparing possession.

#### 1.2.1 Possession constructions

It is possible to compare languages based on the structure and type of their possession constructions. There is not a single way to compare these, as a single construction has many components. One point of comparison is the locus of possession marking in the possession construction (in the sense of head- and dependent-marking, Nichols 1986). Looking just at the locus of marking, some languages have possession constructions that work by simple juxtaposition (as in 3), some mark the possessed noun (as in 4), some mark the possessor, sometimes with a genitive case (as in 5), and some mark both the possessor and possessed noun, as in (6).

(3)

**Table:** 

*yirtyip*	*ŋatan*	*wanaŋgal*	*ŋayi*	
cat	brother	doctor	1sg	
‘My doctor’s brother’s cat’				
(Walsh 1976: 282)				(Murriny Patha, Southern Daly)

(4)

**Table:** 

*Peter*	*’-tul*	
Peter	3-boat	
‘Peter’s boat’		
(Leavitt 1996: 7)		(Malecite-Passamaquoddy, Algic)

(5)

**Table:** 

*pali-eno*	*kala*	
garden-poss	fence	
‘The garden’s fence’		
(Schlatter 2003: 216)		(Tabo, isolate)

(6)

**Table:** 

*sitti-n*	*tinn-issi-n*	*buru*	
lady-gen	3.poss-sister-gen	girl	
‘The lady’s sister’s daughter’			
(Armbruster 1965: 42)			(Kenuzi, Nubian)

One can go into more detail about constructions, such as the presence and type of agreement and the use of different nominal cases (Koptjevskaja-Tamm 2008 gives an in-depth example of this kind of study), or the relative quantity of morphological material in different constructions (as investigated in Nichols 1988).

Further comparisons of constructions can be made by possession class. A claim that inalienable possession involves constructions with less morphological material compares possession constructions by possession classes. However, in this case a definition of alienable and inalienable must first be made using outside criteria.

#### 1.2.2 Syntactic nominal valence

Another way of approaching possession is to ask whether nouns can accept a possessor as a direct modifier. Rather than grouping nouns by constructional profile, this approach is only concerned with the relative obligation of syntactic possession: whether a noun *must*, *may*, or *cannot* have a syntactic possessor.

We will use the term (syntactic) *valence* to describe this approach, keeping it distinct from *lexical* valence or semantic argument structure. By argument structure we mean the lexically specified semantic roles of a verb or noun, while syntactic valence strictly refers to constraints on the syntactic expression of those arguments. In the verbal domain, a word may have a lexically specified semantic role of agent or goal, while having a syntactic expression of subject and object. The semantic argument structure and the syntactic fulfillment of valence have a relationship, but they are not necessarily perfectly aligned (e.g., passivization may change a verb’s valence, but it does not change its semantic arguments).8All syntactic frameworks make this distinction somehow, but our formulation here is similar to that seen in formalisms like Head-driven Phrase Structure Grammar (HPSG), Lexical-Functional Grammar (LFG), and Role and Reference Grammar (RRG), which have an explicit separation between semantic arguments (a semantic and lexical category) and syntactic valence (a syntactic category) (Bresnan et al. 2015; Pollard and Sag 1994; Van Valin 2005).


Possession from the perspective of argument structure leads to a binary distinction between nouns that have a possessor *as a semantic argument* and those that do not. In this view, nouns that lack a possessor as an argument may still have a syntactic possessor, but in this case it is an adjunct (Alexiadou 2003). This distinction in argument structure may or may not be reflected in the grammar through different possession classes, making it difficult to ascertain, and could be driven only by semantics. We therefore will only deal with syntactic valence, since it is definitionally always visible in syntactic constructions.

We extend and adapt our view from Bugaeva et al. (2021), who describe nominal valence chiefly from the perspective of head-marked possession. They describe nouns which always require marking – either indexing a possessor or signaling that no possessor is present (i.e., a marker of un-possession) – as obligatorily possessed, while they describe nouns that never have direct head marking as non-possessible. For example, while most languages would simply omit the possession or person markers to express that the noun is not possessed, Garifuna (Arawakan) nouns of this class require a special non-possession construction in order to be possessor-free, as in (7).

(7)

a.

**Table:** 

*n-anága-n*
1.sg-back-poss
‘my back’

b.

**Table:** 

*anága-ni*	
back-unposs 9	
‘back’ (Haurholm-Larsen 2016: 54)	(Garifuna, Arawakan)

9 We use unposs as a gloss for a marker of un-possession.

There is good reason to generalize the view in Bugaeva et al. (2021) to dependent marking. Languages such as Amarakaeri (Harakmbut) have obligatorily possessed nouns which receive no direct marking, but must have a genitive possessor (8).10That the marker *-edn* is a genitive and not head-marking on the possessed noun can be seen below, where *ndo*ʔ*-edn* means ‘mine’. Example taken from Van linden (personal communication).

(i)

a.

**Table:** 

*mbeʔ-edn*	*ỹã-tã-ẽ*	*in*	*kuwa ?*
who-gen	3sg.dub-appl-be	prox	dog
‘Whose is this dog?’

b.

**Table:** 

*ndoʔ-edn*	*mẽ-tã-ẽ-nẽ*	
1sg-gen	3sg>1/2sg.ind-appl-be-ind	
‘It is mine.’		
(Van linden, pers. comm.)		(Amarakaeri, Harakmbut)

(8)

a.

**Table:** 

*wa-ndik*
unposs-name
‘name’

b.

**Table:** 

*ndoʔ-edn-ndik*	
1sg-gen-name	
‘my name’	
(Van linden 2021: 2)	(Amarakaeri, Harakmbut)

Similarly, in languages like Teko (Tupian) a juxtaposed (compounded) possessor is required. As in Amarakaeri, this is not a head-marking construction.

(9)

**Table:** 

*kunumi-zeburupa*	*am*	
Kunumi-friend	here	
‘Kunumi’s friend is here.’		
(Rose 2011: 186)		(Teko, Tupian)

When classifying the valence of nouns, we consider whether they *can*, *must*, or *cannot* take an adnominal possessor, without the addition of an intervening morphosyntactic head. By an adnominal possessor, we mean that the possessor must be a noun phrase which is a direct syntactic sister of the possessed noun. By a morphosyntactic head, we mean morphemes which project their own node in the syntax, either because they trigger or are the target of agreement, or because they clearly exhibit the properties of an independent verb or noun outside of the possession construction. Such elements could also be defined as non-inflectional (in the sense of Bickel and Nichols 2007), i.e., basic building blocks of syntactic phrases rather than mere reflexes of them. Therefore, possession that is expressed by a relative clause lacks an adnominal possessor in our definition because the possessor functions as the syntactic argument of the subordinated verb, i.e., an intervening head. Likewise, possession that is expressed by possessing a stand-in classifier lacks an adnominal possessor if the classifier serves as a nominal head in the syntax.

##### 1.2.2.1 Optionally possessed

Nouns that may optionally have an adnominal possessor can be viewed as having a valence slot for a possessor which is itself optional.11It is important to note that this optionality only refers to the availability of an adnominal possessor. It is hypothetically possible for a noun to have an optional valence slot, but to have a grammatical requirement that the possessor is expressed somehow (e.g., in a relative clause). Empirically, optionally possessed nouns are by far the most common, as can be seen with the noun *n̓uw̓iiqsu* ‘father’ in Nuuchahnulth (10).

(10)

a.

**Table:** 

*čuu*	*waa=!aƛ*	*n̓uw̓iiqsu=ʔi*
okay	say-now	father=artl
‘“Okay,’ said the father.”		
(Inman, fieldwork notes)

b.

**Table:** 

*n̓amił-šiƛ=!aqƛ=s*	*waa=!at*	*ʔiiqḥuk*	*n̓uw̓iiqsu=ʔak=ʔi*	
try-pf=fut=1sg.strg	say=pass	tell.dur	father=poss=artl	
‘“I will try,’ he told his father.”				
(Inman, fieldwork notes)				(Nuuchahnulth, Wakashan)

##### 1.2.2.2 Obligatorily possessed

Nouns that must have an adnominal possessor are obligatorily possessed. The valence slot of the possessor could be suppressed by the addition of a specific morpheme marking the noun as un-possessed. The noun in this case still has a required valence slot for a possessor, as can be seen by the necessity for overt morphology to suppress it. An example can be seen in Teko, where a prefix *t-* is added when some obligatorily possessed nouns appear without a possessor (11a). Another way for such nouns to appear un-possessed is with a default possessor which does not indicate a specific person. An example can be seen again in Teko, which uses both strategies to unpossess obligatorily possessed nouns (11b).

(11)

a.

**Table:** 

*kob*	*t-apid͡ʒ*	*d͡ʒuriba-we-ʔe*
cop	unposs-house	staircase-also-intens
‘There is a house and a staircase.’		
(Rose 2011: 189)

b.

**Table:** 

*mɨn-a-we*	*zo-ɨpɨ*	*o-pa*	
long.ago-ref-too	indet.ii-ancestor	3.i-be. finished	
‘Long time ago, the elderly died.’			
(Rose 2011: 60)			(Teko, Tupian)

##### 1.2.2.3 Non-possessible

The third possibility is a noun that *cannot* have an adnominal possessor: there is no valence slot for a possessor. A non-possessible noun can only be possessed within a noun phrase by using a subordinate clause (as in Hokkaido Ainu, 12) or classifier construction (as in Ayoreo, 13), if it is even possible to possess it at all.

(12)

**Table:** 

*ku-kor-kur*	*ku-tura*	*Aspet*	*ta*	*arki-as*	
1sg-have-man	1sg-with	name	to	go-1pl	
‘My husband and I went to Ashibetsu.’					
(Shibatani 1999: 36)					(Hokkaido Ainu, Ainu)

(13)

**Table:** 

*j-a-t͡ɕidi*	*tamoko*	
1-thematic.vowel-cl:pet	dog	
‘my dog’		
(Ciucci and Bertinetto 2017: 286)		(Ayoreo, Zamucoan)

As with the other categories (optionally possessed and obligatorily possessed), this is a strictly formal definition, and is not directly related to whether or not it is possible to express ownership. This is only a consideration of the syntactic possibility of adnominal possession.

Syntactic nominal valence is a particular way of interpreting a noun’s constructional profile, but is not the same as a possession class defined by differing constructional profiles. A language may have multiple possession classes which share the same valence type: e.g., a language may have two possessive classes that are both optionally possessed but with different possession constructions (as is the case for Nuuchahnulth), or a language may have multiple possessive classes that are obligatorily possessed but with different un-possession constructions (as is the case for Apurinã). A noun’s valence can always be retrieved from its constructional profile, so long as information about the constructions is sufficiently detailed.

#### 1.2.3 Nominal semantics

Another point of comparison for possession is the *semantics* of nouns that form different possession classes. Possession classes, though defined by their constructional profile, can be compared to each other independently from which constructions they have access to. In the most complete (and in some ways ideal) case, the semantic content of a possession class can be exhaustively defined with a complete list of every noun which belongs to it. These groups can then be abstracted over to various degrees: a set of nouns like ‘father’, ‘mother’, ‘child’, and ‘sibling’ can be collected into an abstract category “nuclear kin”, which could in turn be abstracted into a larger group of “kin”, and so on finally, perhaps, to a category “inalienable”. However, each level of abstraction introduces difficulties for typological comparison. Two languages may differ with respect to whether ‘spouse’ groups together with “nuclear kin” or not. At the highest level, a very abstract “inalienable” class could have radically different (and even non-overlapping) members from one language to another. Every additional semantic abstraction becomes harder to use as a comparandum. However, if there is a universal semantic content behind categories like “inalienable” or “non-possessible” (as suggested in Haspelmath 2017; Nichols 1988), we expect these abstractions to be emergent from finer-grained semantic categories, and ultimately from the entire collection of nouns in the set. In this case one would expect “inalienable” and “non-possessible” to have fuzzy boundaries and be clustered around prototypes.

These three different comparanda for possession – constructions, valence class (the syntactic obligation of a possessor), and semantic content of possession classes – represent different dimensions or views of the possession phenomenon. By independently tracking constructions and possession classes in sufficient detail, we can reconstruct all of these views from the same database.

## 2 Data and methodology

### 2.1 Database overview

Our database contains two distinct types of entries per language: possession classes and constructions. There can be multiple possession classes and multiple constructions for a given language, but we assume that there is always at least one of each (i.e., there is minimally one class of nouns that are all possessed through the same construction). Possession classes and constructions are listed in separate tables with their respective metadata. The two tables are linked with a many-to-many relationship: each construction may be accessible to one or more possession classes, and each possession class may have access to one or more constructions. Each possession class has an ID which is used in the construction table to link an individual construction to the possession class(es) that have access to it. In addition to the possession class table and the construction table, there is also a language table listing the languages and their associated metadata. This language table is linked with the other two tables through a one-to-many relationship using a language ID. The tables and their relationships are described in an associated json file. This database structure follows the CLDF format (Forkel et al. 2018).

#### 2.1.1 Possession Class Table

In the Possession Class Table, each row describes a single possession class of a particular language (see the simplified example in Table 3). The Possession.Class column gives a name for the possession class which is unique for each language. It is used to correlate this table with the Construction Table (see Section 2.1.2), and is not used as an otherwise meaningful label. The Semantic.Categories column lists the categories of nouns present within that particular class, if the class is semantically coherent. Any additional data is provided in the Remarks column, and detailed references are added in the Source column (see Table 3).

**Table 3:** Possession Class Table excerpt.

Glottocode	Possession.Class	Semantic.Categories	Remarks	Source
tomm1242	alienable	default		mcpherson2013tommo:203
tomm1242	inalienable	kin	also includes friend and enemy	mcpherson2013tommo:209
boro1282	alienable	default		crowell1979bororo:214
boro1282	inalienable	body; kin; intimate-property	Based on examples, the inalienables attested in the grammar are: older brother, wife, knife, food, companion, garment, house, bow, hand, father, chief, kinsman, and maybe name.	crowell1979bororo:214
boro1282	non-possessible	animals	The source claims that this class includes domesticated animals giving the examples of chickens, horses and dogs. However, there is no reason to believe that any other animal would be treated differently.	crowell1979bororo:214
ingu1240	alienable	default	There is only a default possession class in Ingush.	nichols2011ingush:592

##### 2.1.1.1 Semantic categories

Possible semantic categories are drawn from an extensible list, initially populated with the semantic categories present in the AUTOTYP database (Bickel et al. 2021). Though there are more categories in our ontology, the semantic categories currently present in our database, as well as their definitions, are listed in Table 4. All semantic categories except the categories *default* and *mixed* have more or less the same content across languages and are therefore comparable. However, some of the categories have looser memberships than others. An extreme example is *intimate_property*, which may contain articles of clothing (including ornaments), tools (including weapons), and one’s own home. The exact set of property terms may differ by language (e.g., whether house or hammer or clothing is included), and so this category is not as narrowly comparable as one like *animals*. The pseudo-categories *default* and *mixed* mark possession classes that are *not* semantically coherent. The category *default* is used for the unique, open, most inclusive possession class of each language. It represents the remainder of the semantic space for a language, when all specially possessed semantic categories have been removed. Its contents can therefore be different across languages. For example, in English, only the category *default* is used, since there is only one class of nouns with regards to possession.12A reviewer points out that there exists literature on possessive differences in English, such as Taylor (1989) and Börjars et al. (2013). However, these observations are dependent on both clinical grammatical acceptability and constructions beyond adnominal possession. Since we restrict ourselves to categorical differences in adnominal possession, we consider English to have a single possession class. In a language with an inalienable class encompassing body parts and kinship terms, the *default* category encompasses all nouns except body parts and kinship terms. The category *mixed* is used for possession classes that are closed, but not semantically coherent. A language may have more than one *mixed* possession class, but only one *default* class.

**Table 4:** Semantic categories used in this study.

Semantic category	Definition
animals^a^	animals, wild or domesticated
wild_animals	wild animals only
body^b^	body parts in a broad sense, possibly including mental faculties, feelings, name, etc., as well as (some) body functions, excreta, footprints, etc.
body_internal	internal body parts only
humans	human or higher animate (divine or supernatural beings, etc.)
kin	a kin relation
blood_kin	a kin related by blood (not marriage)
nature_inanimate	rocks, mountains, celestial objects, etc.
nuclear_kin	only parents, siblings, and children
owner	‘master’, ‘owner’, etc.
part	the part in part-whole relations; spatial relations nouns, locatives, etc.; subsetting terms like ‘group’, ‘part’, etc.
plant_part	plant parts (root, leaf, branch, etc.)
place_rel	native land, village, etc.^c^
plants	whole plants and types of plants
intimate_property	furniture, tools, weapons, clothes, ornaments
names	person and place names
mass_noun	all uncountable nouns
mixed	a lexically specified class with no semantic cohesion
default	the default set of nouns, membership in which is determined once all other semantic categories are subtracted^d^

^a^Of the 12 languages that list animals among their non-possessible nouns, only one source for Bororo (Bororoan) specifies that these are domesticated animals (Crowell 1979). However, we do not believe that the non-possessibility of domestic animals entails that wild animals are possessible. We think it far more plausible that in Bororo *all* animals are non-possessible, and that possession almost always indicates domestication. This analysis is strengthened with data from Teko (Tupian), where the classifier construction used to possess animals can be used for typically domesticated animals (chickens, dogs) and for wild animals that are exceptionally held as pets (frog, tapir, Rose 2011, Rose p.c.). Similarly in Kakua (Kakua-Nukak), the default possessive construction is acceptable with animals only if raised as pets or captured as prey (Bolaños 2016). We have therefore not considered domesticated animals as one of our semantic categories. However, we have included wild animals as a semantic category, since when grammars specify that wild animals are non-possessible, it is very possible that domesticated animals are treated differently, e.g., according to the default possession strategy. ^b^Initially, we had a *body* and a *body_extended* category, but we found essentially no language that didn’t have at least some “extended” terms, such as feelings, human attributes, name, spirit, etc., so we ended up merging them. Many grammars do not explicitly describe the behavior of such nouns, so this is a category that could be underrepresented in our study, if it was separate. ^c^The noun “house” is commonly encountered as a member of the inalienable class, and it could be conceptually categorized as *place_rel* or *intimate_property*. We have categorized it on a language-by-language basis depending on what other nouns are present in the same possession class. ^d^In some cases, this category may include a body part possessed not by its inherent possessor.

#### 2.1.2 Construction Table

In the Construction Table, each row describes a construction in a particular language (see the simplified example in Table 5). The Construction.Form column contains a relatively abstract representation of the construction. It has some standard slot representations, such as PSSR and PSSD for “possessor” and “possessed”, as well as language-specific material, such as the form or gloss of specific morphemes. For example, the genitive construction in English could be represented as [PSSR*=s* PSSD] or [PSSR=GEN PSSD]. The main purpose of the construction form is to facilitate recognition of the construction when consulting resources for the language. The construction form abstraction level is not standardized and is not used further in the database or our analyses. The Construction.Type column is a fine-grained classification of possessive constructions and will be described further in Section 2.1.2.1. Any automatic treatment of our construction data is based on the construction type,13Exceptions here are the special possession and unpossession constructions that have [NULL] for their construction form. See Section 2.1.2.2 for more details. which is highly standardized. The Possession.Class column lists the possession class(es) that have access to the construction as a possessed noun. Finally, there is a Remarks column for additional information, and a Source column with detailed references.

**Table 5:** Construction Table excerpt.

Glottocode	Construction.Form	Possession.Class	Construction.Type	Remarks	Source
tomm1242	PSSR PSSD{Low Tone NP}	alienable	PSSD	For alienable possessed nouns, low tone is induced in the whole NP including modifiers.	mcpherson2013tommo:204
tomm1242	PSSR PSSD{Low Tone N}	inalienable	PSSD	For inalienable possessed nouns, low tone is induced only on the possessed noun, and not on modifiers.	mcpherson2013tommo:210
boro1282	PSSR person.set2 PSSD	alienable	PSSD[AGR:PSSR]	This is the 2nd paradigm of possessive markers in the grammar used for all nouns except animals and inalienable nouns. It looks a lot like a classifier construction and historically most likely was, but there is no synchronic evidence that the presumed classifier functions as a noun.	crowell1979bororo:216
boro1282	PSSR person.set1-PSSD	inalienable	PSSD[AGR:PSSR]	This paradigm of possessive markers is the same as that used for verbal indexation.	crowell1979bororo:215
boro1282	PSSR person.set3 PSSD	non-possessible	PSSD[AGR:PSSR]	This is the 3rd paradigm of possessive markers in the grammar used only for animals. It looks a lot like a classifier construction and historically most likely is, but there is no synchronic evidence that the presumed classifier functions as a noun.	crowell1979bororo:216
ingu1240i	PSSR.GEN PSSD	alienable	PSSR[AGR:PSSR]	The possessor is in genitive case. The genitive suffix is not segmented in the grammar, and it is different depending on the number of the possessor.	nichols2011ingush:448, 127

##### 2.1.2.1 Construction Types

Constructions in the Construction Table are assigned to different types according to a combination of criteria: the syntactic relationship between possessor and possessed noun, presence and locus of marking, and presence and patterns of agreement.14In this paper, we use the term “agreement” in a loose sense, without necessarily assuming an overt noun with which the morpheme agrees. Our separation of locus of marking and agreement is exactly analogous to the separation of marking and indexing described in Evans and Fenwick (2013). Excluding agreement, we recognize the following hierarchy of types:

– Aggregate type direct: the possessor is strictly adnominal, i.e., without intervening syntactic heads:
  – Type juxt: the possessor and the possessed noun are juxtaposed or compounded.15While juxtaposition and compounding may be distinguishable in principle, this is not always the case and it can be difficult with old sources and underdocumented languages.
  – Type marker: there is at least one *inflectional* marker that encodes a possession relationship in addition to the two nouns.
  – Subtype pssr: marking occurs on the possessor only.
  – Subtype pssd: marking occurs on the possessed noun only.
  – Subtype pssr+pssd: marking occurs on both the possessor and possessed noun.
  – Subtype linker: Neither possessor nor possessed noun are marked, but there is an inflectional linker which does not clearly form a syntactic constituent with only the possessor or only the possessed noun.
– Aggregate type indirect: the possessor can be expressed adnominally only by adding an intervening head, i.e., an independent, non-inflectional, and/or open-choice, lexical element to the construction:
  – Type class: there is a nominal classifier as intervening syntactic head and it is necessary for expressing possession.
  – Type clause: there is a subordinate verb as an intervening head and it is necessary for expressing possession.

These main construction types are refined further with the addition of information about agreement patterns, as in example (14) from Burushaski (isolate). In Burushaski, for inalienable nouns, both possessor and possessed noun are marked. Also, both markers agree with the possessor: the possessor marker, a genitive, agrees in gender and number,16In Burushaski there is a genitive form for female human singular nouns, and another form for all other nouns. and the possessed marker agrees in person, gender, and number (Munshi 2018). This construction has been assigned the type pssr[agr:pssr]+pssd[agr:pssr]. In order to increase consistency and efficiency in construction type assignment, we are using a detailed key17A key, or more precisely an identification key, is a common tool used in systematic biology to identify organisms. It is usually dichotomous (each question/step in the key has two possible answers/outcomes). It must be noted here that a key is not a claim as to which construction types are more similar to each other. A similar device has been used in children’s literature for stories that have multiple possible evolutions and endings (choose-your-own-adventure). that can be found in the Supplementary Materials. This decision-making tool distinguishes the most common types and allows more complicated or unanticipated cases to be identified and discussed further.

(14)

a.

**Table:** 

*t* ^*h*^ *am-* ***e***	***i*** *-riŋ-aṭe*	*booza-an*	*del-imi*
king- **gen.default**	**3m.sg**-hand-on	kiss-sg.ndef	hit-pst.3m.sg
‘[…] he kissed the king’s hand.’			
(Munshi 2018: 256)

b.

**Table:** 

*in-* ***mo***	***mu*** *-uyar*	*ooɣatanas-an*	*bai*	
3sg- **gen.f.sg**	**3f.sg**-spouse	teacher-sg.ndef	be.pres.3m.sg	
‘[…] her husband is a teacher.’				
(Munshi 2018: 123)				(Burushaski, isolate)

As previously mentioned in Section 1.2.2, our definition of a possessive classifier construction requires that the classifier function as a nominal head in the morphosyntax. In classical phrase-structure grammar, this means that the classifier has a corresponding node in the tree with its own part-of-speech information. Though this determination has to be made holistically, there are a few tests which can signify that an element is a syntactic nominal head. In the absence of evidence of this kind, we do not categorize a construction as belonging to type class and treat the morpheme in question as inflectional. In more detail, these tests are:

1. Lexical independence: If an element can serve as a regular noun outside of a possession construction, then we treat it as projecting a head in the possession construction as well.
2. Lexical choice: If the selection of a classifier is open to speaker choice and not grammatically determined (e.g., a selection between long.skinny and large), then it carries lexical information and is a nominal head.
3. Agreement: If the classifier triggers agreement (e.g., it causes agreement in adjectives or on the verb) or is the target of agreement (e.g., it receives plural marking to agree with the possessed noun), then it is a head. But care must be taken in this case to differentiate the potential classifier from the possessed noun.
4. Verb selection: If the classifier is targeted by verbs to receive case marking or a specific adposition, then it is a head. As with the above, care must be taken to differentiate the classifier from the possessed noun.

As an example of a construction categorized as class, take the possession of non-relational nouns (the default class) in Xavánte (Nuclear-Macro-Je, cf. 15). This construction uses the morpheme *te*, which does not occur elsewhere as a free word. However, it can be shown that *te* functions as a syntactic nominal head, since it, rather than the notionally possessed noun, takes any case marking required by the verb (16). Consequently, we consider this a classifier construction.

(15)

**Table:** 

*warazu*	***te***	*ubu*	*wa-te*	*ʔmadöʔö*	*zaʔra*	*mono*	*õ*	*di.*	
white.man	**rgn**	face	1pl.erg-aux	3.abs.see	pl.disc	iter	neg	expl	
‘We had not seen the white man’s face.’									
(Machado Estevam 2011: 380)									(Xavánte, Nuclear-Macro-Je^18^)

18 Special glossing for Xavánte: ego ‘egophoric’, expl ‘expletive’, iter ‘iterative’, pl.disc ‘discrete plural’, rgn ‘relational generic noun’.

(16)

a.

**Table:** 

*ĩĩ-mama*	***te***	***ma***	*wapsã*	*wa*	*tãma*	*ti-a*
1sg-father	**rgn**	**dat**	dog	ego	3.dat	3.abs-give
‘I gave (food to eat) to my father’s dog.’

b.

**Table:** 

*ĩĩ-mama	***te***	*wapsã*	***ma***	*wa*	*tãma*	*ti-a*	
1sg-father	**rgn**	dog	**dat**	ego	3.dat	3.abs-give	
Intended: ‘I gave (food to eat) to my father’s dog.’								
(Machado Estevam 2011: 408)							(Xavánte, Nuclear-Macro-Je)

In Bororo (Bororoan), there are three series of person markers used in possessive constructions: a series of bound pronominals which are used in verbal indexation as well as for inalienable possession, and two other series used for alienable possession and animal possession respectively (Table 6). Nominal possession is expressed with the appropriate possessor-indexing element accompanied by a preceding possessor, as in (17) (Crowell 1979).

**Table 6:** Bororo bound pronominals (used in inalienable possession), and person markers used in alienable and animal possession (Crowell 1979: 216).

Person	Bound pronominal	Alienable	Animal
1	i-	ino	inagu
2	a-	ako	akagu
3	*∅*-/u-	o	aku
coreferential	tɨ-/xi-	to-	tagu
reciprocal	pu-	pu-	pugagu
1pl incl	pa-	pago	pagagu
1pl excl	xe-	xeno	xenagu
2pl	ta-	tago	tagagu
3pl	e-	eno	enagu

(17)

**Table:** 

*José*	*aku*	*kowaru*	*redoku-re*	
José	3sg.animal	horse	run-neutral	
‘José’s horse ran.’				
(Crowell 1979: 82)				(Bororo, Bororoan)

The bound pronominals are apparently related to the forms of the other two paradigms. The paradigms for alienable and animal possession could be understood either as independent paradigms with different grammatical marking or as the bound pronominals *i(n)-*, *ak-*, *∅* *-*, etc. affixed to seperate classifiers *o and *aku. However, there is no evidence of the lexical independence of hypothetical classifiers *o and *aku, and we cannot perform the other tests, as Bororo has no case, and plurals and postpositions are expressed at the end of the noun phrase. Though it appears quite plausible (indeed, likely) that the system seen in Table 6 developed from two independent classifiers,19We think that classifier constructions more generally can grammaticalize into either marking that attaches to the possessor (pssr type) or marking that attaches to the possessed noun (pssd type), which indicates possession becoming more syntactically direct. We leave further exploration of this grammaticalization pathway for future work. from the data available to us, the elements *-o* and *-aku* do not appear to function this way synchronically.

##### 2.1.2.2 Special construction types

Apart from possessive constructions, there are a number of special constructions included in the Construction table. They enable us to describe accurately the differential behavior of Possession Classes with respect to possession.20These special construction types and the associated special construction form [NULL] are used in our automated methods for treating construction data. These include:

– *Unpossession construction*: The construction employed when a noun is not possessed. Usually, this is a construction of just the bare noun [N], and in this case it is not entered in the database. However, there are cases of nouns that require a special marker in order to appear unpossessed or with an unknown possessor. Such constructions are included in the database and are marked as unpossession for their construction type.
– *Null unpossession construction*: If a noun class is claimed to always appear possessed, we represent it as having access to a construction of the unpossession type with the form [NULL].
– *Null possession construction*: In some languages, a noun class is claimed to be unpossessible under any circumstances. This is represented in the database with a construction that has possession as its type and [NULL] as its form.

### 2.2 Language sample

For this study we employed a phylogenetically diverse global sample of 20 languages per macroarea (Hammarström and Donohue 2014), for a total of 120 languages. All the languages included in our sample belong to different families and are a subset of the languages included in the Possession Module of the ATLAs database (Inman et al. in prep). The full sample and the data are available in the Supplementary Materials. We follow Glottolog (Hammarström et al. 2021) for language names and classifications.

### 2.3 Methods of analysis

Our database can be used to examine possession with various approaches. We have already introduced three different possible views of possession, based on constructions, valence, and semantics. In this section we will briefly explain how we arrive at each of these views using our database. Then we will present the analyses performed and the questionnaire used for a short possession survey. We used custom scripts written in R (R Core Team 2021) to validate data consistency and to perform quantitative analyses (for more information see the Supplementary Materials).

#### 2.3.1 Arriving at different views of possession

The construction and semantic views are directly accessible in the database through the Construction and the Possession Class tables respectively. In the Construction Table, each construction is directly associated with the possession class(es) that have access to it. In the Possession Class Table, each possession class is directly associated with the semantic categories belonging to this class.

The valence view is not directly encoded in the database but it can be derived using the construction table. We can determine the valence of the nouns belonging to each class as follows:

– *Non-possessible* nouns have access *only* to possession constructions of the indirect aggregate type, or a special NULL possession construction (in this case, the nouns cannot be possessed at all). They do not have any unpossession construction.
– *Obligatorily possessed* nouns have access to at least one possession construction of the direct aggregate type *and* to a special unpossession construction (which may be [NULL]).
– *Optionally possessed* nouns have access only to direct possession constructions and they do not have access to a special unpossession construction; or they have access to both direct and indirect constructions.21If a noun can be both directly and indirectly possessed, it is optionally possessed from a valence perspective, even if it has an unpossession construction, thus rendering part of the first condition irrelevant.

#### 2.3.2 Semantic network visualization

In order to investigate the cross-linguistic evidence for the categories commonly called “inalienable” and “non-possessible”, we visualize all semantically coherent possession classes (i.e., with most members belonging to one or a few semantic categories) of all languages in our sample in a semantic network. To do this we need to partition the semantic space in categories applicable to all languages.

We started from the semantic categories we found relevant for our data, which have been already presented in Table 4. Some of these categories are nested within other categories, as is the case for *body_internal* (covering internal organs), which is a proper subset of *body*. In order to devise a set of non-overlapping semantic categories, we organized these attested semantic categories into a partially hierarchical structure, as can be seen in Table 7. Each semantic category on the left contains the semantic categories to its right. The rightmost categories comprise the set of non-overlapping semantic categories used for crosslinguistic comparison. The pseudo-categories *mixed* and *default* are not included in this structure, since they are both used for semantically non-coherent classes.22There is an additional category, *mass_noun*, that is not included in the semantic hierarchy and the semantic network visualization. This category was found in only one language, Negidal, where the same possession construction is used for typically non-possessible nouns (names, animals, natural phenomena) and all mass nouns (Pakendorf and Aralova, this issue). Although there is a semantic content to the category of mass nouns, it is a cross-cutting category with respect to all others, and it would complicate the overall picture.


**Table 7:** Hierarchy of semantic categories.

kin	blood_kin	nuclear_kin
		DD_non_nuclear_blood_kin
	DD_non_blood_kin	
part	plant_part	
	DD_other_part	
	body	body_internal
		DD_body_external
nature	plants	
	nature_inanimate	
	animals	wild_animals
		DD_domestic_animals
	humans	
relation	owner	
	place_relation	
	DD_other_relation	
intimate_property		
names		

Due to the nested nature of our original semantic categories, among the resulting non-overlapping categories there are some that are never attested, as they represent the remainder of a more inclusive category when a nested category is subtracted. In our previous example with *body_internal* and *body*, once *body_internal* is subsumed under *body*, a new category *body_external* is implied. We call such semantic categories “deduced” and they are preceded by “DD” in Table 7. When constructing the semantic network, each high level category is replaced with all the non-overlapping categories it contains.

**Figure 1:**
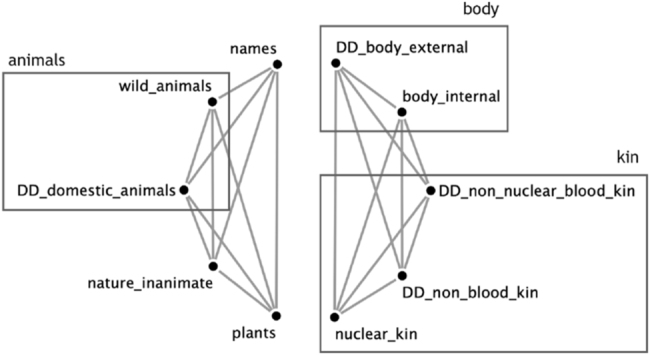
The full network of semantically coherent categories in Hixkaryána.

All non-overlapping semantic categories are potential nodes of a comprehensive semantic network. A coherent semantic class can then be represented with edges connecting the semantic categories it contains. For example, Hixkaryána (Cariban) has two semantically coherent possession classes: one containing body parts and kinship terms, and the other animals, plants, natural phenomena and proper names (Derbyshire 1979). Plotting the two semantically coherent classes of Hixkaryána results in the networks in Figure 1. We then repeat this process for every semantically coherent class in every language and we overlay the resulting networks. For the visualization we used Cytoscape v.3.9.0 (Shannon et al. 2003) and the aMatReader application (Settle et al. 2018).

#### 2.3.3 Survey questions

In this section we present the questionnaire used to query our database of 120 languages for a short survey on possession worldwide. Our questionnaire can be divided in two parts: questions 1–4 are basic exploratory questions for the phenomenon of differential possession across languages, while questions 5–11 investigate the characteristics of particular classes based on their semantics.

As we will see in the results section, the semantic network visualization resulted in two well-separated networks of conceptually inalienable (mostly body parts and kinship terms) and conceptually non-possessible (mostly animals, humans and natural elements) possession classes, which allowed us to use these two broad types as a basis for further comparison. Thus, for the second set of questions, we grouped together all classes of the type “conceptually inalienable” and “conceptually non-possessible.”

The full list of survey questions used can be seen below:

1. How many possession classes are there?
2. How many semantically coherent possession classes are there?
3. What is the valence of nouns in the default class?
4. How are nouns of the default class possessed?
5. How many conceptually inalienable classes are there?
6. How many conceptually non-possessible classes are there?
7. What is the valence of nouns in conceptually inalienable classes?
8. How are nouns in conceptually inalienable classes possessed?
9. How are nouns in conceptually inalienable classes unpossessed?
10. What is the valence of nouns in conceptually non-possessible classes?
11. How are nouns in conceptually non-possessible classes possessed?

## 3 Results and discussion

### 3.1 Semantic network results

**Figure 2:**
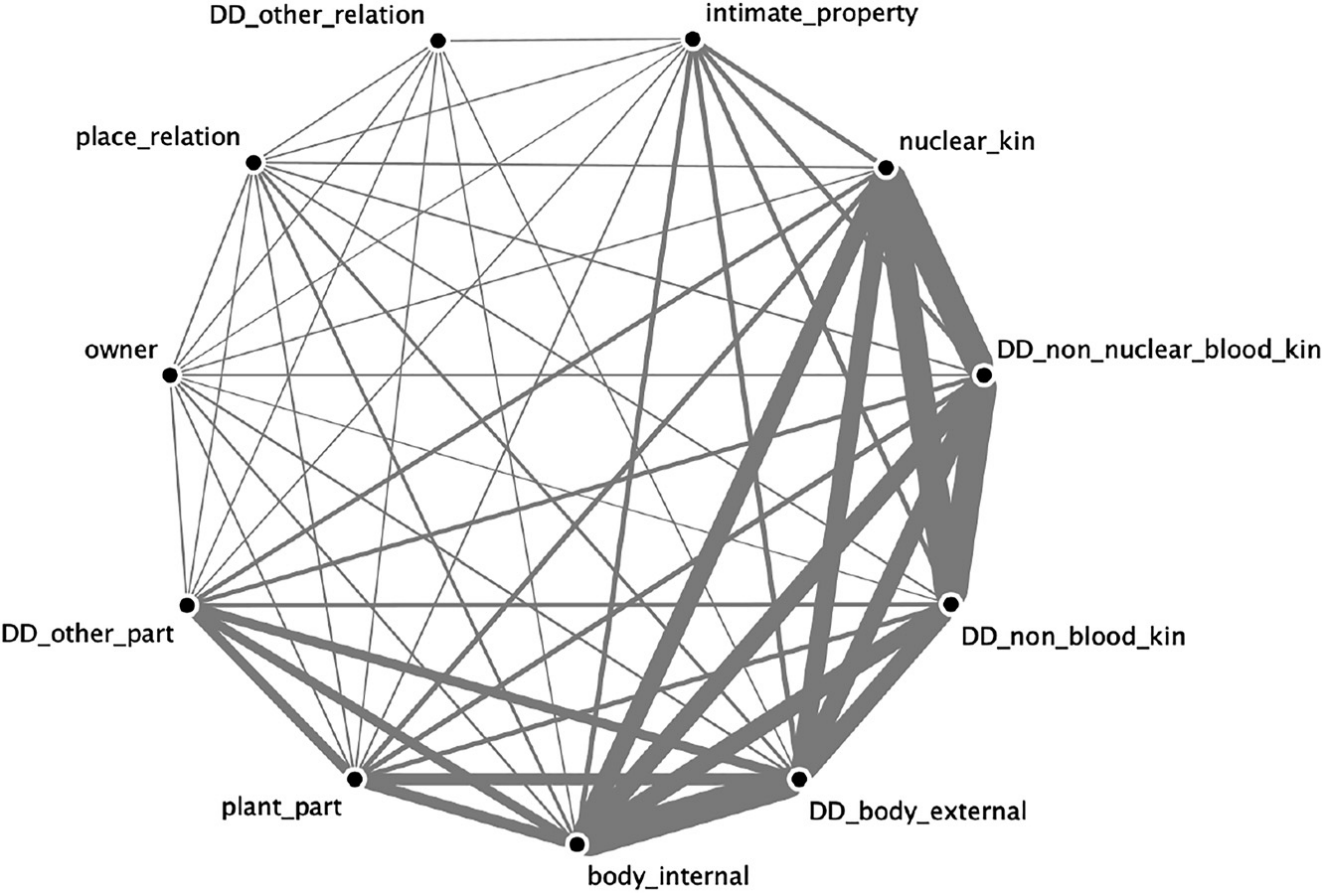
The network of conceptually inalienable classes.

**Figure 3:**
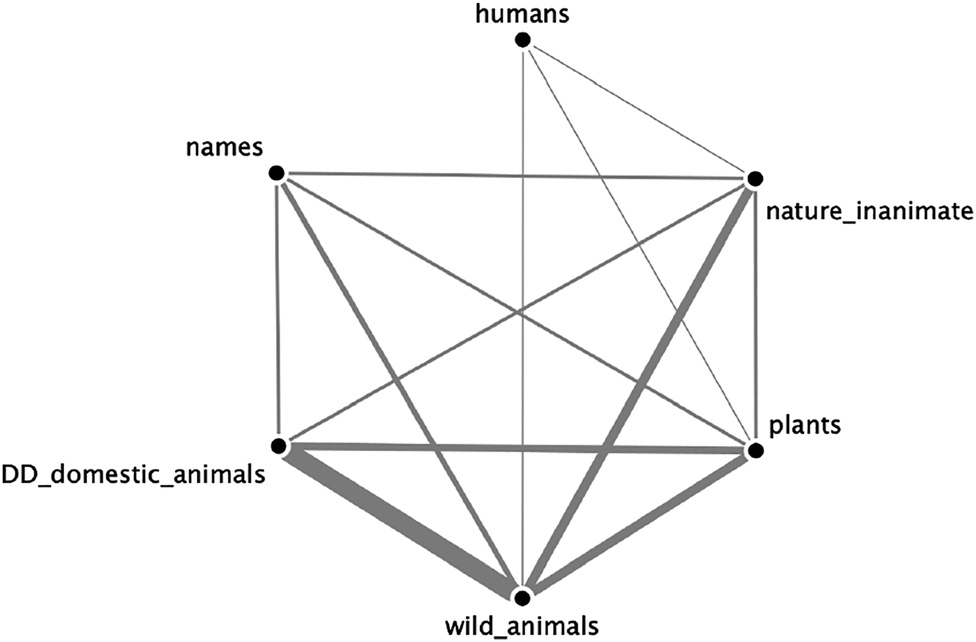
The network of conceptually non-possessible classes.

The result of the overlay of all semantically coherent possession classes attested in our data revealed two unconnected networks, which are given separately in Figures 2 and 3. Relative thickness of edges represents the number of connections between semantic categories (i.e., frequency that two semantic categories are found in the same semantically coherent possession class).

The fact that these two networks remain totally unconnected means that not a single language in our sample of 120 has a semantically coherent possession class that contains a semantic category from each of the two networks. We do not mean to imply that such an overlap is necessarily impossible, but it empirically must be rare. This also does not mean that no single semantic concept appears in both networks, but that if it does it must be exceptional and not categorical, as described in Section 1.1.2. We have termed these non-overlapping semantic networks “conceptually inalienable” (Figure 2) and “conceptually non-possessible” (Figure 3). This finding supports linguist intuitions about the semantic correlates of possession types.

The conceptually inalienable nouns have a clear core membership of kinship and body part terms, a more distant set of part-whole relations (including plant parts), intimate property, and finally a more peripheral set of members like owner and place relationships (homeland, village, etc.). These are all united by the notion of *unchosen* or *inherent* relationship, with kin and body parts the most stereotypical: one has the body parts one has and cannot un-have them except through violence, and one similarly does not choose who one’s kin are and cannot have that relationship sundered except perhaps by a (socially) violent break. This is true for part-whole relations as well (such as the leaf of a plant, or the top of a table), the notable difference being that these are both non-human and inanimate. Finally, the notion of an inherent relationship can extend to clothing, tools, relational places like village, homeland, origin, and so on.

Conceptually non-possessible nouns have a core membership of animals, with plants and inanimate nature (rocks, earth, sun, etc.) more distant from this core, and finally humans and names (or proper nouns) the most distant. Like conceptually inalienable nouns, there is an underlying notion shared among conceptually non-possessible classes: none of these nouns are really owned in a “state of nature.” Animals are only owned in the context of domestication or captivity, where human intervention has altered the (conceptual) state of nature. Similarly, plants are not owned in their natural habitat, and nor are people, except by some (perhaps socially) violent action that forces an ownership relation. This weakly extends to named entities, which are always uniquely identifiable, and are prototypically people or important entities held in common.23It must be noted here that most grammars do not mention if and under what conditions proper nouns can be possessed. It may well be that names of entities are non-possessible in many more languages than appear in our results. This is the exact opposite of the conceptually inalienable semantic classes, which in their state of nature are either parts of a whole or stand in an intimate relationship with something else.

Although there are clear semantic and conceptual correlations with these classes, the full reasons for their separation cannot be determined only from a typological study. These two semantic collections could represent something about how human beings inherently conceptualize the world, they could represent cultural developments of integrating items into the human sphere of control (Bugaeva et al. 2021), either independently and in parallel or through shared histories, or this could be a by-product of the total frequency with which different types of possessive relations are expressed (Haspelmath 2017), or any combination of these factors. Regardless of their ultimate source, the two semantic clusters are empirically distinct and can be used for further research. Every semantically coherent possession class in our sample can be unambiguously categorized as either belonging to the conceptually inalienable network or the conceptually non-possessible network. We have therefore used these two types as a basis of further comparison.

### 3.2 Survey results

In this section we present summary statistics based on the survey questions, beginning with results on a per-language basis, followed by results on a per-possession-class basis.

#### 3.2.1 Results by language

Table 8 shows how many languages have a certain number of classes of a particular type. For instance, every language has exactly one default class, so the “Default” row shows that all 120 languages have just one default class. The number of conceptually inalienable classes is more varied: in 57 languages, there are no classes of this semantic type, in 51 languages there is exactly one class of this semantic type, in 10 languages there are exactly two classes of this type, and so on. As this table shows, about half of the languages in our sample (54 out of 120) have only a single possession class (the default class), while the rest (66 out of 120) have more than one possession class (i.e., an (in)alienability contrast). The vast majority of non-default possession classes are semantically coherent. Only five languages in our sample have semantically non-coherent but closed (our pseudocategory *mixed*) possession classes: Apurinã (Arawakan), North Slavey (Athabaskan-Eyak-Tlingit), Highland Oaxaca Chontal (Tequistlatecan), Sandawe (isolate), and Maricopa (Cochimi-Yuman).

**Table 8:** Number of languages with a particular number of classes.

	Number of classes									
	0	1	2	3	4	5	6	7	8	9
Default	0	120	0	0	0	0	0	0	0	0
Inalienable	57	51	10	1	1	0	0	0	0	0
Non-possessible	111	8	1	0	0	0	0	0	0	0
Mixed	115	3	0	1	1	0	0	0	0	0
Total possession classes	0	54	47	13	3	2	0	0	0	1

Among the languages that have two possession classes, the vast majority have a conceptually inalienable class in addition to their open default class (45 out of 47). Only two languages with only two possession classes have a conceptually non-possessible class in addition to the default class: Yuracaré (isolate), and Mohawk (Iroquoian). A small part of our sample has three possession classes (13 out of 120). Of them, five have a conceptually inalienable and a conceptually non-possessible class, seven have two conceptually inalienable classes, and one has a conceptually inalienable class and a *mixed* class. Finally, there are a small number of languages with more than three possession classes: three languages with four (Highland Oaxaca Chontal [Tesquistlatecan], Limilngan [Limilngan-Wulna], North Slavey [Athabaskan-Eyak-Tlingit]), two languages with five (Teko [Tupian] and San Dionisio del Mar Huave [Huavean]), and one language with nine (Apurinã, Arawakan). Among these languages with a high number of possession classes, two (Limilngan and Teko) have only semantically coherent classes in addition to their *default* class, one (Highland Oaxaca Chontal) has only *mixed* classes, while three (North Slavey, San Dionisio del Mar Huave, and Apurinã) have both semantically coherent and *mixed* classes. The highest number of semantically coherent possession classes present in a language is four and is attested in Teko and Apurinã. These two languages also have the highest number of conceptually non-possessible and conceptually inalienable classes in our sample: Apurinã is the only language with four conceptually inalienable classes, while Teko is the only language with two conceptually non-possessible classes.

It is interesting to note that many of the “extreme” cases in our sample are languages from the Americas. All of the languages in our sample with *mixed* or semantically non-coherent possession classes are American. Both languages which have a conceptually non-possessible class without a conceptually inalienable class are American, and of the nine languages with a conceptually non-possessible class, seven are in the Americas (the exceptions being Grass Koiari and Negidal). This correlates highly with non-possessible valence: of eleven languages where the phenomenon is observed, only three are outside of the Americas (Grass Koiari, Ainu, and Abun). All but one of the six languages with four or more possession classes are American. This does not mean that complex systems cannot be found elsewhere. Bickel and Nichols (2013) find the largest number of possessive classes among their survey in Amele, a language of Papua New Guinea, and they report additional cases of languages with non-possessible classes outside the Americas. Nevertheless, the frequency with which American languages (in comparison with other macroareas) in our sample exemplify these properties – large numbers of possession classes, non-possessible classes, and *mixed* semantics – is notable.

#### 3.2.2 Results by possession class

Every language has by definition one default class, which we use as a basis of comparison across languages. However, some languages have more than one conceptually inalienable class, while one has two conceptually non-possessible classes. In order to describe the main characteristics of conceptually inalienable classes cross-linguistically, we have pooled all such classes from all languages together, for a total of 78 conceptually inalienable possession classes. We have done the same with conceptually non-possessible classes (a total of 10 classes) and mixed classes (also a total of 10). Statistics for this cross-linguistic pooling of possession classes are given for possession constructions in Table 9, for un-possession constructions in Table 10, and for valence classes in Table 11. In the aforementioned tables we include results for mixed classes for completeness, but since they are not cross-linguistically comparable, we will not comment upon them further.

**Table 9:** Available possession constructions for conceptual classes.

	MARKER	JUXT	CLAUSE	CLASS	NULL	MARKER & JUXT	MARKER & CLAUSE	Sum
Default	97	11	4	0	0	7	1	120
Inalienable	51	15	1	0	0	10	1	78
Non-possessible	2	0	0	2	6	0	0	10
Mixed	9	0	0	0	0	1	0	10

**Table 10:** Unpossession constructions for conceptual classes.

	Noun	MARKER	NULL	Sum
Default	120	0	0	120
Inalienable	47	15	16	78
Non-possessible	10	0	0	10
Mixed	7	3	0	10

**Table 11:** Valence of conceptual classes.

	Optionally possessed	Obligatorily possessed	Non-possessible	Sum
Default	116	0	4	120
Inalienable	47	30	1	78
Non-possessible	2	0	8	10
Mixed	7	3	0	10

##### 3.2.2.1 Default classes

When comparing the unique open (pseudo-category *default*) possession class across languages, we can see that it contains overwhelmingly optionally possessed nouns. However, there are four languages whose default possession class is comprised of non-possessible nouns: Wichita (Caddoan), Maricopa (Cochimi-Yuman), Hokkaido Ainu (Ainu), and Abun (isolate).

With respect to construction types, the majority of languages with optionally possessed default nouns (97 out of 116) use a construction of the type marker (head marking, dependent marking, double marking, linkers), while a sizable minority (11 out of 116) use a construction of the type juxt (juxtaposition, compounding). The remaining languages with a default class of optionally possessed nouns use two construction types: either juxt and marker or clause and marker (see Table 9). All four languages with non-possessible default nouns use subordinate clauses (type clause) to possess them.

##### 3.2.2.2 Conceptually inalienable classes

About one third of the conceptually inalienable classes in our sample consist of obligatorily possessed nouns (30 out of 78), while the rest are optionally possessed nouns. There is only one language, Wichita (Caddoan), which uses clauses to obligatorily possess its conceptually inalienable class (the same construction it uses to possess its default class). In other words, the conceptually inalienable class of Wichita is non-possessible on the valence view. We propose to call such a case non-possessible but obligatorily owned.

By definition, obligatorily possessed nouns either have access to a special unpossession construction or they can never be unpossessed (corresponding to our special [NULL] unpossession construction). These two cases are attested in equal proportions in our sample (15 each). The construction types used for the possession of conceptually inalienable classes (with the exception of Wichita as mentioned above which uses clause) are marker (51 out of 78) and juxt (15 out of 78), while there are ten cases where both these construction types can be used for the same conceptually inalienable class, and one case where both marker and clause constructions are available.

##### 3.2.2.3 Conceptually non-possessible classes

Conceptually non-possessible classes are much less common in our sample (a total of 10). Among them, eight consist of syntactically non-possessible nouns, while two languages, Negidal (Tungusic) and Bororo (Bororoan), have a conceptually non-possessible class of optionally possessed nouns. For six of the conceptually non-possessible classes the sources claim that the relevant nouns can never be possessed (i.e., our [NULL] possession construction). The rest can be possessed either through an indirect construction (Yuracaré [isolate] and Teko [Tupian]) or through a direct construction (Negidal and Bororo). Both cases with indirect constructions involve possessive classifiers (type class) rather than clauses (type clause).

## 4 Conclusions

In this work we have presented a database structure that encodes a high-resolution representation of differential possession. By treating possession classes and possessive constructions as independent but linked, our database can accommodate possession classes that are semantically coherent or not, as well as a many-to-many relationship between possession classes and possessive constructions. Relatively rare phenomena, such as conceptually non-possessible classes, or syntactically non-possessible default classes can be incorporated seamlessly into this coding scheme. We have presented a semantic ontology, as well as a construction categorization scheme, both of which are extendable. Finally, our database can be used to investigate the relationships between the semantic, constructional, and valence dimensions of differential possession cross-linguistically.

We have exemplified our methodology with a phylogenetically diverse global sample of 120 languages and presented preliminary results on the semantic dimension of possession classes and how it correlates with noun valence and possessive strategies. Our semantic network analysis confirms the long-standing linguists’ intuition of the universality of conceptually inalienable nouns (Heine 1997), but also confirms a universal core of non-possessible nouns as well (Bugaeva et al. 2021; Haspelmath 2017). Conceptually inalienable nouns include body parts and kinship terms at their core, but they commonly extend to include plant parts or parts in general, intimate property, such as tools and clothing, as well as relational nouns, such as owner, friend, or homeland. Conceptually non-possessible nouns on the other hand have animals at their core, but they often include humans, plants, natural phenomena and inanimate objects, as well as proper names. Conceptually non-possessible noun classes are rare cross-linguistically and it seems possible that both their presence and their semantic correlates have been under-reported.

Based on our survey results, we conclude that possession classes are relatively common and they typically are semantically coherent. When two possession classes are present in a language, it is much more common that the closed class is conceptually inalienable, rather than conceptually non-possessible. When three semantically coherent possession classes are present in a language, the two closed classes could be either both conceptually inalienable, or one conceptually inalienable and the other conceptually non-possessible. Languages with more than 3 possession classes are rare. Default classes consist overwhelmingly of optionally possessed nouns, but cases of non-possessible default classes are attested. Dependent-marking, head-marking, and juxtaposition are all common possession strategies, while default non-possessible classes are typically possessed using subordinate clauses. Among conceptually inalienable classes, it is about twice as likely that, in terms of valence, the nouns are optionally possessed rather than obligatorily possessed. Juxtaposition seems more common among conceptually inalienable classes than default classes. Finally, conceptually non-possessible classes usually consist of syntactically non-possessible nouns. In some cases, ownership of conceptually non-possessible nouns can be expressed with a classifier or dedicated head-marking construction.

Further research is needed to examine in more detail how semantic categories cluster in possession classes, as well as the association of different semantic categories with noun valence and possessive strategies. For instance, by associating construction types to conceptual classes (rather than the other way around), we can quantitatively test the claim that inalienable possession classes are more likely to have less morphological material than alienable possession classes. Another promising avenue of investigation is to take full advantage or our construction types to explore how different variables such as locus of marking and presence and patterns of agreement correlate with other properties of possession classes. Finally, we are interested in investigating areal patterns arising from nominal possession, in terms of the semantic categories that are specially possessed, the constructions available to the language, and also valence patterns.

The methods introduced here represent a first step in a broader program of creating a multi-variate typology of possession. Our results have already demonstrated the usefulness of this approach, and we hope our methodology will prove fruitful not only for possession, but also as an inspiration for the study of other multi-dimensional linguistic phenomena.

## Supplementary Materials

Supplementary Materials for this paper can be found at the OSF repository: https://osf.io/zurd6/.
